# Numerical and Experimental Investigation of Morphological Modification on Fused Silica Using CO_2_ Laser Ablation

**DOI:** 10.3390/ma12244109

**Published:** 2019-12-09

**Authors:** Li Zhou, Youen Jiang, Peng Zhang, Hui Wei, Wei Fan, Xuechun Li, Jianqiang Zhu

**Affiliations:** 1 National Laboratory on High Power Laser and Physics, Shanghai Institute of Optics and Fine Mechanics, Chinese Academy of Sciences, Shanghai 201800, China; zhoul@siom.ac.cn (L.Z.);; 2 University of Chinese Academy of Sciences, Beijing 100049, China

**Keywords:** laser polish, laser ablation, morphological modification, fused silica

## Abstract

In this paper, a numerical model based on the finite-element method for predicting the morphological evolution during CO_2_ laser ablation on fused silica is developed and examined experimentally. Adopting the optimized parameters that were obtained from the model, a typical cone-shaped multi-stage structure with a diameter of 2 mm and a slope angle of 10.4° was sufficiently polished. Both the roughness and the transparency of the surface structure were significantly improved. The characterized slope angle of the continuous surface is exactly consistent with the predicted value, and the ablation depth is 32 ± 1.247 µm with a deviation of 1.7% (RMS, root mean square). The deviation is principally caused by the neglect of melting displacement in simulation and the irregularity in actual stepping structures. These results indicate that the numerical model can simulate morphological modification of CO_2_ laser ablation with a high degree of reliability. It could further be used to optimize processing parameters for customizing continuous fused silica surfaces, which could facilitate industrial manufacturing of freeform optics.

## 1. Introduction

Laser processing is an effective and attractive method for surface modification of various materials, including surface texturing of semiconductors [1,2], corrosion prevention in biocompatible metals [3,4], and surface polishing of optical glass [5,6]. Employing CO_2_ laser melting in a non-evaporative regime has enabled us to obtain surfaces of optical glass with a roughness smaller than 0.1 nm at a rapid processing rate of up to 5 cm^2^/s [5]. In the process, mass transportation and surface reshaping are predominantly driven by viscous and thermal capillary forces [7]. This intrinsic mechanism suggests that high-quality surface modification using laser melting is mainly restricted to a small spatial range with a fluctuation height of less than 1 µm [8,9] and a spatial wavelength of less than 100 µm [5].

Fortunately, for fluctuation with a spatial wavelength larger than 100 µm, Weingarten et al. [5,6] introduced the laser-beam figuring (LBF) process to correct errors in the shape of a polished surface of fused silica with a precise and selective material ablation using a pulsed CO_2_ laser. In the LBF process, laser ablation is highly localized and the ablation depth is strongly related to the pulse duration, which allows for the elimination of a wide fluctuation (over 100 µm). Meanwhile, the method of the transverse scanning of laser pulses does not cause deterioration in the peripheral areas, such as an increase in micro-roughness or thermal residual stress. Therefore, we believe that surfaces with high fluctuation (beyond 1 µm) could be similarly processed by longitudinal accumulation of numerous pulses with an interval for sufficient cooling. This approach gives us the opportunity to convert an arbitrarily etched stepping structure into a continuous one. However, the achievement of continuous optical surfaces with the highest precision by laser-pulse ablation, namely, the optimum morphological modification, requires a balance of several parameters, including pulse energy, repetition frequency, pulse duration, spot dimension, and interval between adjacent spots. An analytical model developed by Nowak et al. [10] can be used as a guide for parameter selection in CO_2_ laser ablation of fused silica; however, comprehensively predicting the morphology of a polished workpiece remains highly difficult. In order to rapidly obtain the optimum parameters for state-of-the-art polishing, it is essential to develop a numerical model to predict the structural deformation of fused silica under different laser-ablation conditions.

Here, we systematically investigated the morphological modification (polishing) of a multi-stage structure on fused silica using CO_2_ laser ablation. Primarily, a numerical model based on the finite-element method was established to predict the morphological evolution of the structure under different laser-heating conditions. Subsequently, by utilizing optimum parameters that were acquired from the numerical model, a typical multi-stage structure that was initially manufactured by ultrafast laser inscription was processed via CO_2_ pulsed laser ablation. Finally, the morphology of the processed structure was observed and characterized, and the measurement was found to be in agreement with the simulation results.

## 2. Materials and Methods

Fused silica, Borofloat^®^ 33, and BK7 are three types of commonly used optical glasses with different SiO_2_ compositions. The CO_2_ laser processing parameters significantly vary according to the different optical and thermal properties of glasses in different compositions. Optical constants determine the fraction of laser irradiation that is required to heat a glass and the region of the heat-affected zone on the glass. BK7 has a higher reflectivity and larger absorption coefficient than fused silica at the wavelength of interest of 10.6 µm [11]. Working conditions for processing glass are strongly influenced by the viscosity and thermal shock resistance of the glass. For instance, BK7 has the lowest processing temperature among the three types of glass because it displays the fastest decline in viscosity with increasing temperature. Additionally, Borofloat^®^ 33 and BK7 often undergo fractures, so extra preheating is necessary to reduce the thermal shock during CO_2_ laser processing [5,12]. In contrast, fused silica allows for processing without preheating due to its higher thermal shock resistance. Therefore, fused silica is a better candidate for achieving higher surface precision, because the experimental error resulting from the preheating process can be avoided in this case.

**Figure 1:**
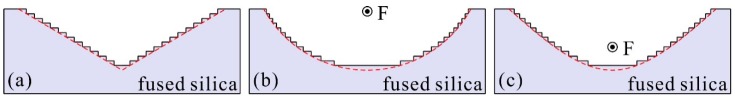
Cross-section views of different quasi-continuous structures. (**a**) A conical surface; (**b**) a cylindrical surface; (**c**) a parabolic surface. Red dashed lines represent the desired continuous profiles for special applications. Point F in (**b**) and (**c**) stands for the focal point of the surface.

Fused silica (Corning, New York, NY, USA) samples with a diameter of 25.4 mm and a thickness of 5 mm were used in the investigation of morphological modification. Arbitrary quasi-continuous structures, such as conical, cylindrical, and parabolic surfaces, on a sample were inscribed by an ultrafast laser (Light Conversion, Vilnius, Lithuania, Pharos) layer-by-layer as a preliminary measure. These multi-stage structures for further polishing using CO_2_ laser ablation are not continuous and typically have a step height of ~1 µm, as depicted in Figure 1. Since multi-stage structures in diverse shapes are similar except for different step widths, a conical structure was selected as the representative for investigation in the following stages.

### 2.1. Numerical Model of CO_2_ Laser Ablation

**Figure 2:**
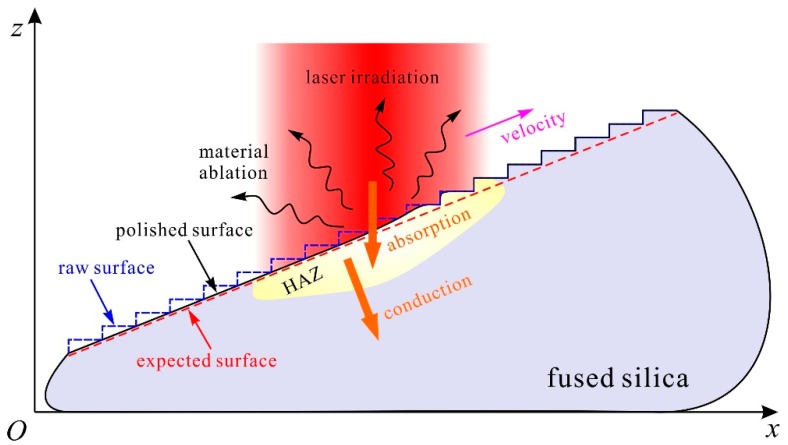
Schematic diagram for a two-dimensional (2D) numerical model of CO_2_ laser ablation.

A limited heat-affected zone (HAZ) based on laser ablation allows for a precise polishing process on a multi-stage structure, which yields the arrangement of thousands of laser pulses for polishing a customized structure. To optimize parameters for processing a multi-stage structure, a numerical model based on the finite-element method was developed for analyzing thermal conduction and structural deformation during the laser-ablation process. In non-explosive laser ablation with laser intensity in the regime of ~0.1–1 MW/cm^2^, material removal by evaporation dominates, and uncontrollable melt displacement and ejection are avoidable [10,13]. Additionally, the pulse repetition frequency under investigation here is less than 2 kHz, which allows for enough cooling time between pulses to avoid the effect of heat accumulation [14]. Given the symmetry of a conical multi-stage structure, it is possible to simplify the polishing process into a two-dimensional model, as schematically illustrated in Figure 2. A stepwise raw surface is drawn as the initial surface for laser ablation. The temperature at the sites absorbing the incident laser irradiation increases sharply to the vaporization threshold of ~3500 K at normal atmospheric pressure [10], which initiates material evaporation over microsecond time scales. According to the Hertz–Knudsen–Schrage formula, in the case of lossless, uniform laser irradiation of an absorbent, semi-infinite solid, the velocity of surface recession *υ_e_* can be described as [10,15]

(1) $$v_{e}=I_{p}\frac{1-R}{\rho \Delta H},$$

where *I_p_* = *I*_0_ exp[−2(x − *υt*)^2^/*ω*_0_^2^] *f*(*t*) is the incident laser intensity at position x and time frame *t*, *I*_0_ is the axial intensity of a Gaussian spot, *v* is the velocity of the spot, *ω*_0_ is the radius of the spot at 1/e^2^, *f*(*t*) represents the square wave with a pulse duration of τ and a pulse repetition of f_r_, *R* is the reflectivity at the wavelength (0.15 @ 10.6 µm), *ρ* is the density of fused silica (2201 kg/m^3^), and Δ*H* is the total change in enthalpy required to volatilize the material (11.4 MJ/kg) [10,16,17]. If the laser spot on the raw surface of a multi-stage structure is scanned, then the surface will gradually become continuous in the designed shape. The parameters that were used for the simulation are shown in Table 1. It should be mentioned that the diameter of the Gaussian spot was set to 120 µm@1/e^2^, which is equal to the value in the experimental system.

### 2.2. Experimental Setup

**Figure 3:**
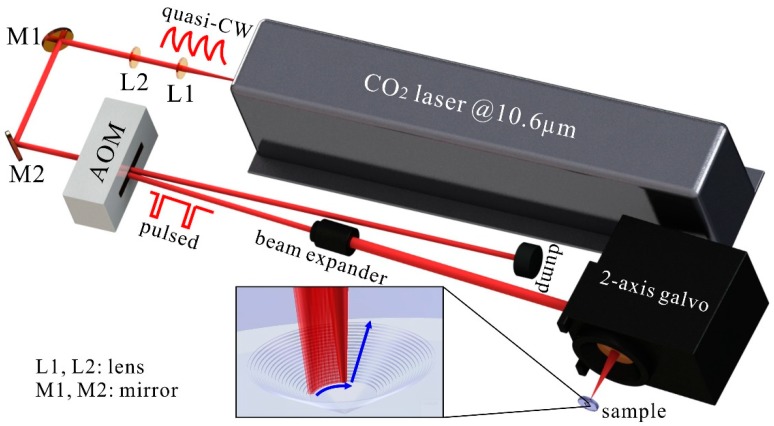
Experimental setup for surface modification by pulsed laser ablation. The enlarged view is a quasi-conical multi-stage structure under CO_2_ laser polishing, where the blue arrows indicate the direction of the movement of the laser spot.

Figure 3 illustrates the experimental setup for surface modification by pulsed laser ablation. A CO_2_ laser (Synrad, Mukilteo, WA, USA) operating at 10.6 µm was employed to polish the surfaces of the multi-stage structure on a fused silica substrate because of the high absorption at this wavelength. The quasi-continuous wave emitted from the laser was chopped into approximately square pulses with a repetition frequency below 2 kHz and a duration in the microsecond regime, which was accomplished by an acousto-optic modulator (Isomet, Manassas, VA, USA). After traveling through a pair of collimated lenses and a beam expander, the beam was focused into a small spot (120 µm@1/e^2^) using a ZnSe f-θ lens with a 75 mm focal length. A sample was mounted on a motorized linear stage to precisely position the surface of interest at the focal plane. The position of the focal spot in the focal plane was controlled by a two-axis galvo (Scanlab, Puchheim/Munich, Germany, intelliSCAN) to complete the pre-designed motion patterns. In the case of polishing a quasi-conical multi-stage structure, the focal spot was moved along a series of concentric circles until the whole morphological modification process finished, as indicated in the insert in Figure 3.

## 3. Results and Discussion

### 3.1. Prediction of Morphological Modification

**Figure 4:**
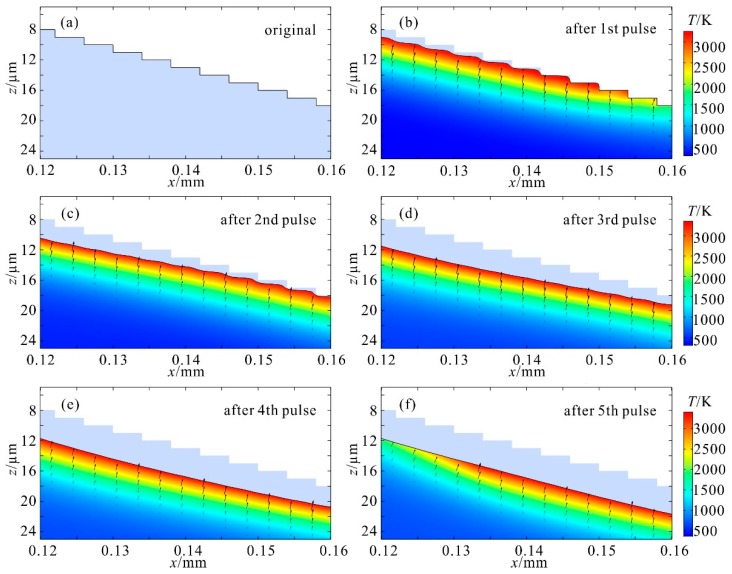
A morphological evolution and temperature profile during a complete polish process (f_r_ = 1 kHz, τ = 10 µs, d_i_ = 20 µm). (**a**) The original multilevel structure; surface profile at the end of the (**b**) first, (**c**) second, (**d**) third, (**e**) fourth, and (**f**) fifth pulses, compared with the original shape in light purple in each figure. Color maps in (**b**–**f**) are temperature profiles.

A typical structural evolution and temperature profile during the smoothing process is displayed in Figure 4. The laser spot with a diameter of 120 µm was moved from the position of x = 0.1 mm to x = 0.18 mm with a velocity of 20 mm/s. Considering a complete polish cycle, the zone exhibited in the figure is an area irradiated by all five pulses. Shapes in light purple in these figures are the original multi-stage structures. The boundaries of the color maps are surface profiles at different time frames during the process. Compared with the original structure, it is clear that stairs are in gradual recession under the irradiation of successive laser pulses. Furthermore, for every cycle of irradiation, the sharp edges are always subjected to ablation first, then the subjected area became wavelike, as shown in Figure 4b. Owing to the non-uniform heating inherent to the laser-pulse ablation, the spatial distribution of the laser intensity results in the variation in surface temperature, leading to the spatial non-uniformity in ablation depth. It was not until the end of all of the five laser pulses that the stepwise structure turned into the expected continuous slope without waviness, as shown in Figure 4f.

**Figure 5:**
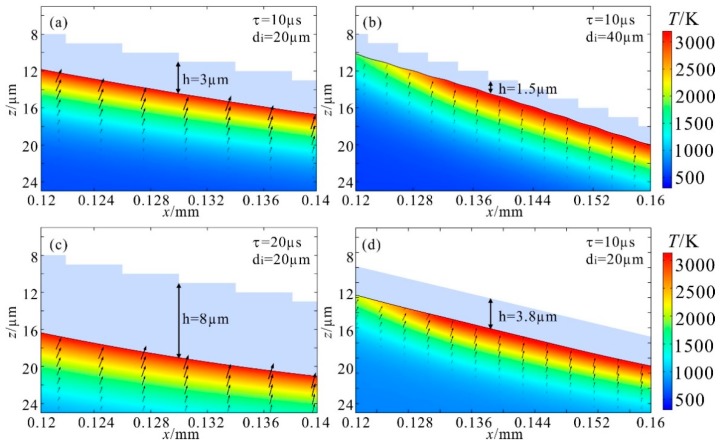
Typical morphological modification. (**a**) τ = 10 µs, d_i_ = 20 µm; (**b**) τ = 10 µs, d_i_ = 40 µm; (**c**) τ = 20 µs, d_i_ = 20 µm; (**d**) τ = 10 µs, d_i_ = 20 µm. The area in light purple is the original profile. The color maps present the temperature profiles at the end of a pulse.

Comparatively, it is preferable to acquire a polished profile that is closer to the expected one with less material loss, which means a shorter ablation depth is more desirable. Primarily, pulse energy is usually selected to be slightly higher than the ablation threshold for achieving a rapid and non-explosive evaporation, which depends on the pulse duration. As a compromise between polishing efficiency and heat accumulation during the process, the pulse repetition frequency was calculated by simulation to be 1 kHz. After determining the pulse energy, repetition frequency, and spot dimension, the effects of pulse duration in the regime of 10~100 µs on the morphological modification can be investigated. With a longer pulse duration, the ablation depth is deeper and the polished profile curves more. Figure 5a,c are typical profiles produced by pulse durations of 10 µs and 20 µs, respectively. Apparently, the profile polished with a pulse duration of 10 µs is straight and has an ablation depth of 3 µm, while profile polished with a pulse duration of 20 µs becomes somewhat curved and has a deeper ablation depth of 8 µm. It can be inferred that a shorter pulse duration is better for morphological modification, but the pulse energy was unable to reach the ablation thresholds with a pulse duration shorter than 10 µs in the experiment. Therefore, 10 µs should be the optimum pulse duration in this case.

Based on the optimized parameters, cases with different intervals between adjacent spots were simulated. The results show that when the interval is larger than 20 µm, the stepwise structure becomes wavelike, indicating that inadequate polishing dominates except for in sites near the center of the laser spot. Representatively, when the interval extends to 40 µm, in spite of a half ablation depth, as illustrated in Figure 5b, the profile is wrinkled rather than straight. When the interval narrows down, for instance, to 10 µm, the morphology remains a straight slope but the ablation depth increases. Consequently, an interval of 20 µm is preferred for the morphological modification.

Considering that an actual multi-stage structure manufactured by an ultrafast laser should have slightly irregular, instead of uniform, steps, a repetitive process is needed to sufficiently polish the structure. Thus, morphological modification of a straight slope processed by the optimum parameters of τ = 10 µs, d_i_ = 20 µm was simulated as well. As illustrated in Figure 5d, the slope has a uniform decline of 3.8 µm, with no sign of surface distortion. This indicates that if a structure were polished several times, there would be no effect on the slope angle, though the ablation depth would increase by some factor. Comprehensively, for a three-dimensional conical structure of rotational symmetry, the optimum parameters are a pulse duration of 10 µs and an interval of 20 µm between adjacent spots. It should be mentioned that the interval between adjacent spots involves the step between two successive shots and the space between two traces of laser spots in close proximity. Thus, the laser spot should be moved along a series of concentric circles with diameters increasing by 20 µm at a speed of 20 mm/s at the repetition frequency of 1 kHz.

### 3.2. Experimental Verification

In order to verify the developed numerical model, multi-stage conical structures were processed using the experimental setup of CO_2_ laser ablation with the wavelength at 10.6 µm. The multi-stage conical structures with identical parameters were prepared on a fused silica sample by ultrafast laser inscription with the equipment described in [18]. Initially, laser ablation with the optimum parameters that were obtained from the numerical model was performed on the multi-stage structure to eliminate the stages and generate a continuous slope. Subsequently, the morphology of both the original multi-stage structure and the processed one were measured using an optical imaging assembly (Navitar, Rochester, NY, USA), a stylus profiler (Bruker, Tucson, AZ, USA), and a cold field emission scanning electron microscope (Hitachi, Tokyo, Japan, SU8220).

**Figure 6:**
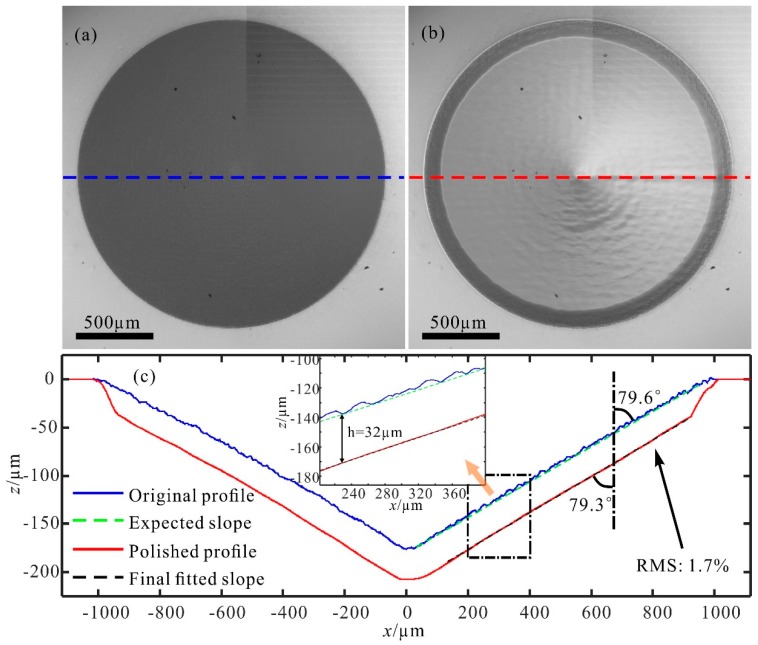
Optical microscope images of (**a**) the original multi-stage structure and (**b**) the structure after laser ablation. (**c**) Cross-section views of the multi-stage structure before and after polishing. The insert is an enlarged view of the area in the rectangle. Profiles are characterized by a stylus profiler with a vertical (z-direction) resolution of 8 nm and a transversal (x-direction) resolution of 0.1 µm. Slopes (dashed lines) were fitted according to the profiles.

The original conical multi-stage structure manufactured by an ultrafast laser has a rough surface that scatters most incident light and appears to be opaque, as shown in Figure 6a. However, it becomes as transparent as the substrate after modification by CO_2_ laser ablation, as seen from Figure 6b. The manufactured rough structure with a diameter of 2 mm was characterized by the stylus profiler, which has a vertical (z-direction) resolution of 8 nm and a transversal (x-direction) resolution of 0.1 µm, and is fitted with an angle of 79.6°, i.e., a slope angle of 10.4°, as shown in Figure 6c. It is visible that the original structure has stairs as high as ~8 µm, which is beyond the ablation depth of one set of the series of concentric circles with optimum parameters. Hence, we processed it with eight sets of concentric circles to thoroughly polish all the stairs. The profile of the polished structure was measured with an angle of 79.3°, a difference of 0.3° from the expected slope, as indicated in Figure 6c. Clearly, all stairs of the original structure disappeared, so the surface became smooth and continuous. The roughness Ra has a significant reduction from 1.899 µm to 0.475 µm. It should be mentioned that the polished structure has an ablation depth of 32 ± 1.247 µm, which is almost equal to the predicted value of 29.6 µm (3 µm + 7 × 3.8 µm). The deviation of ablation depth is 1.7% (RMS, root mean square) except for the bottom and edge of the concave cone. Therefore, the proposed model is proven to be valid in surface prediction and effective in optimizing process parameters for a customized structure.

**Figure 7:**
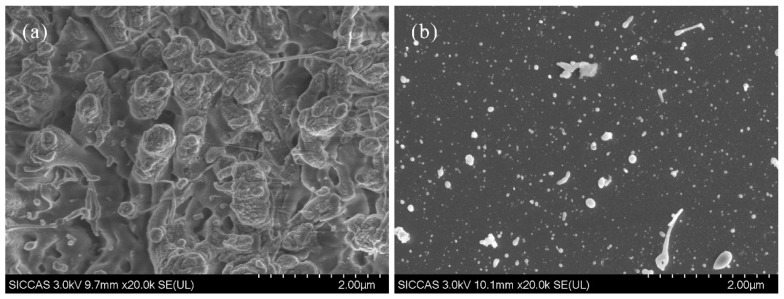
Typical SEM images at the slope of (**a**) the original multi-stage structure and (**b**) the structure after laser ablation.

Additionally, Figure 7 displays the SEM images of the slope area of a typical original multi-stage structure and its counterpart after laser ablation. Disorderly particles and gullies of various dimensions in the original rugged surface disappear after the ablation, and its microstructure evolves to become more uniform and with fewer and finer particles. This suggests the excellent quality of the processed surface.

**Figure 8:**
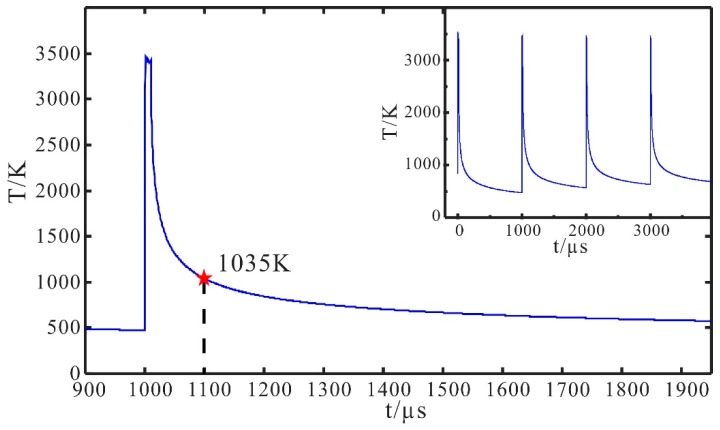
Axial-temperature beyond a laser pulse. The red star indicates the temperature of 1035 K at 100 µs after the start of a laser pulse. The insert displays the typical variation of the axial-temperature during the pulsed laser-ablation process.

With regard to the difference between the theoretical prediction and experimental outcome, it may result from the surface relaxation in a thin melt layer below the ablated zone that was neglected in the simulation and the irregularity of the original multi-stage structure manufactured by an ultrafast laser. Surface relaxation in the thin melt layer includes a minor structural deformation driven by viscous and capillary forces before cooling down [7,14] and densification in depth of several dozen nanometers due to rapid cooling from a high temperature [19,20]. Figure 8 illustrates the axial temperature beyond a laser pulse during the ablation process, which was calculated based on the developed numerical model. At the end of each pulse during the smoothing process, the temperature remains above the melting points and then sharply declines to 1035 K within a timescale of 100 µs.

## 4. Conclusions

A two-dimensional finite element model was developed to analyze the heat transmission and mass transportation during the process of CO_2_ laser polishing using pulsed laser ablation, which was proved to be well consistent with the experimental results and effective in predicting morphological modification. Based on the numerical model, parameters having impacts on the final profile were investigated and the optimum processing condition for polishing a typical conical multi-stage structure was attained after balancing the shape error and mass loss. Using the optimized parameters, a concave cone with a slope angle of 10.4° and a diameter of 2 mm was polished and found to be of excellent quality, with an ablation depth of 32 ± 1.247 µm. The deviation of the ablation depth is 1.7% (RMS). In contrast to the non-evaporative method, which limits the polish depth within the melt layer, the pulsed laser ablation can polish structures with step heights largely beyond the absorption depth.

Pulsed laser ablation is also applicable to multi-stage structures in shapes of paraboloid, cylinder, or other customized surfaces. Combined with ultrafast laser inscription, it would be able to manufacture highly precise continuous freeform optics, which are widely used to compensate for optical aberrations in state-of-the-art image systems. More accurate prediction of the numerical model requires a more comprehensive consideration in further work, such as using the measurement of a fabricated multi-stage structure as the input, and taking account of the effects of material melting.

## Figures and Tables

**Table 1:** The parameters that were used in the simulation based on the 2D numerical model of CO_2_ laser ablation.

Parameter	Value
pulse energy E	0.5–5 MJ
pulse repetition frequency f_r_	0.5–2 kHz
pulse duration τ	10–100 µs
diameter of focus spot 2ω_0_	120 µm@1/e^2^
interval between the adjacent spots d_i_	10–120 µm
